# Heterozygous *SERPINA1* Defects and Their Impact on Clinical Manifestations of Patients with Predominantly Antibody Deficiencies

**DOI:** 10.3390/ijms25105382

**Published:** 2024-05-15

**Authors:** Styliani Sarrou, Ioanna Voulgaridi, Athanasia Fousika, Katerina Dadouli, Olympia Margaritopoulou, Ioannis Kakkas, Christos Hadjichristodoulou, Fani Kalala, Matthaios Speletas

**Affiliations:** 1 Department of Immunology & Histocompatibility, Faculty of Medicine, University of Thessaly, 41500 Larissa, Greece; ssarrou@uth.gr (S.S.); afousika@uth.gr (A.F.); omargarit@uth.gr (O.M.); fkalala@uth.gr (F.K.); 2 Laboratory of Hygiene and Epidemiology, Faculty of Medicine, University of Thessaly, 41222 Larissa, Greece; ioavoulg@uth.gr (I.V.); adadouli@uth.gr (K.D.); xhatzi@uth.gr (C.H.); 3 Department of Immunology and Histocompatibility, “Evaggelismos” General Hospital, 10676 Athens, Greece; ioankakkas@evaggelismos-hosp.gr

**Keywords:** predominantly antibody deficiencies, common variable immunodeficiency, alpha-1 antitrypsin, *SERPINA1*, liver disease

## Abstract

Patients with predominantly antibody deficiencies (PADs) display hypogammaglobulinemia with a high prevalence of infections, along with autoimmune manifestations, benign and malignant lymphoproliferation and granulomatous disease. It is noteworthy that PAD patients, even those with defects in the same causative genes, display a variable clinical phenotype, suggesting that additional genetic polymorphisms, located in either immune-related or non-immune-related genes, may affect their clinical and laboratory phenotype. In this context, we analyzed 80 PAD patients, including 70 with common variable immunodeficiency (CVID) for *SERPINA1* defects, in order to investigate the possible contribution to PAD clinical phenotype. Ten CVID patients carried heterozygous pathogenic *SERPINA1* defects with normal alpha-1 antitrypsin levels. Interestingly, the presence of the Z allele (rs28929474), which was found in three patients, was significantly associated with liver disease; hepatic complications were also observed in patients carrying the p.Leu23Gln (rs1379209512) and the p.Phe76del (rs775982338) alleles. Conversely, no correlation of *SERPINA1* defective variants with respiratory complications was observed, although patients with pathogenic variants exhibit a reduced probability of developing autoimmune diseases. Therefore, we recommend *SERPINA1* genetic analysis in PAD in order to identify patients with a higher risk for liver disease.

## 1. Introduction

Predominantly antibody deficiencies (PADs) represent the most common type of inborn errors of immunity (IEIs) in humans, characterized by a wide variation in disease onset, clinical manifestations and outcome [1,2]. Among PADs, common variable immunodeficiency (CVID) is the most prevalent disorder. CVID is characterized by permanent and sustained hypogammaglobulinemia (concerning all immunoglobulin isotypes as a rule), absent isohemagglutinins, poor responses to vaccines and a high prevalence of infections, along with autoimmune manifestations, benign and malignant lymphoproliferation and granulomatous disease [3,4].

Genetic defects resulting in CVID are obscure for many cases, since only 14 types of CVID due to monogenic defects have been described in the Online Mendelian Inheritance in Man (OMIM) database to date (https://www.omim.org/entry/ accessed on 21 March 2024). Some of these defects appear to predominantly affect the disease phenotype, while they may be present in otherwise healthy individuals, as has been established, for example, for *TNFRSF13B/TACI* defects (CVID2, OMIM # 240500) [5,6]. Moreover, some patients with a CVID-like clinical phenotype display only combined IgA and IgG subclass deficiencies; genetic defects are also obscure for the majority of affected patients [7,8]. Finally, during recent years, in some patients with an initial diagnosis of CVID, *CTLA4* mutations were identified as the causative defect, leading to the reclassification of their condition as immune dysregulating syndrome [9,10]. Patients with CTLA4-mediated disease exhibit hypogammaglobulinemia along with autoimmune manifestations, lymphadenopathy and/or inflammatory bowel disease [9,10]. However, patients with PAD—also including patients with defects in the same causative genes—display a variable clinical phenotype suggesting that additional genetic polymorphisms, located in either immune-related or non-immune-related genes, may affect their clinical and laboratory phenotype.

Alpha-1 antitrypsin (AAT) encoded by the *SERPINA1* gene, is a member of the serine protease inhibitor (serpin) superfamily. It is primarily produced in the liver and plays a crucial role in protecting the lungs from a serine protease, the neutrophil elastase, during inflammation caused by infections or irritants like tobacco smoke [11,12,13]. The deficiency in AAT, known as alpha-1 antitrypsin deficiency (AATD), predominantly affects the lungs, resulting in early onset emphysema and chronic obstructive pulmonary disease (COPD) [12,14]. Moreover, depending on the *SERPINA1* defect that can lead to abnormal AAT polymerization and intracellular protein accumulation in liver, affected patients may develop liver dysfunction, eventually progressing to liver cirrhosis [14].

Apart from its antiprotease activity, AAT displays additional biological effects, including the ability to modulate both inflammation and apoptosis [11,13]. In this context, recent studies suggest that AAT has immunomodulatory effects and may play a role in the emergence and/or management of autoimmune disorders. For example, therapies based on AAT are being explored in patients and animal models for their potential to treat various autoimmune diseases—such as type 1 diabetes, systemic lupus erythematosus, and rheumatoid arthritis—by modulating the immune response [15]. However, the relationship between AATD and autoimmunity is complex and still under investigation.

A limited number of studies have been conducted in patients with PAD to explore the role of AAT and AATD in disease phenotype, with inconclusive results [16,17,18]. PAD can contribute to lung damage over time due to acute or chronic infections, which may affect the respiratory system [19,20]; moreover, some patients may develop liver disease, which is usually associated with infections and autoimmunity [21,22]. Consequently, the aim of our study was to investigate the possible contribution of *SERPINA1* defects in the clinical phenotype of patients with PAD, influencing the severity and the progression of their disease.

## 2. Results

### 2.1. Overview of Clinical Characteristics of Study Patients

An overview of patients’ clinical characteristics is presented in Table 1. Considering the manifestations related to common clinical effects of AATD, we recorded that 25 patients (31.3%) suffered from chronic respiratory disease (including 13 with chronic restrictive pulmonary disease (CRPD), 7 with COPD, and 5 with combination CRPD and COPD). Moreover, eleven patients (13.8%) presented with liver disease, including two patients with nodular regenerative hyperplasia (NRH), three patients with granulomatous disease (one also developed cirrhosis), two patients with unexplained elevated transaminase levels (one eventually developed cirrhosis), a patient with primary biliary cirrhosis, a patient with overlap syndrome (autoimmune hepatitis and primary biliary cirrhosis), and two patients with cirrhosis after chronic hepatitis B virus (HBV) and hepatitis C virus (HCV) infection, respectively.

### 2.2. SERPINA1 Defects in the Study Patients

*SERPINA1* genetic analysis revealed both pathogenic and non-pathogenic defects in 67 out of 80 participants (84.0%) (Table 2). Among them, ten patients (12.5%) exhibited pathogenic defects, all in a heterozygous state; interestingly, all these patients suffered from CVID, and their clinical characteristics are detailed in Table 3. The most common defect, found in four individuals, was a substitution of cytosine by thymine at nucleotide 1177 (c.1177C>T, rs61761869), resulting in a change of a proline to a threonine (p.Pro393Thr). The second most prevalent defect, found in three individuals, was the common variant of the Z allele of the protein (c.1096G>A, p.Glu366Lys, rs28929474). Three additional pathogenic *SERPINA1* defects were detected in three female patients: the common variant of the S allele (rs17580, c.863A>T, p.Glu288Val); rs1379209512 (c.68T>A, p.Leu23Gln); and rs775982338 (c.226_8delTTC, p.Phe76del). CVID patients with heterozygous *SERPINA1* defects were analyzed for AAT serum levels, and all displayed levels in the normal range.

Moreover, non-pathogenic *SERPINA1* defects were observed in study patients. The most prevalent was the missense mutation characterized by the M1A allele (rs6647, c.710T>C, p.Val237Ala), which was found in 35 patients (43.7%; 32 in a heterozygous state and 3 in a homozygous state). Additionally, 30 patients (37.5%; 26 in a heterozygous state and 4 in a homozygous state) carried the M3 allele (rs1303, c.1200A>C, p.Glu400Asp), and 24 patients (30.0%; 21 in a heterozygous state and 3 in a homozygous state) carried the M2/M4 allele (rs709932, c.374G>A, p.Arg125His). Additional non-pathogenic defects were also found in a lower frequency, as presented in detail in Table 2.

Interestingly, we identified a novel intronic defect in a heterozygous state (c.1066-87T>C) in a female patient with *CTLA4*-mediated immune dysregulation syndrome, without lung or liver disease. Moreover, two sisters with CVID displayed another very rare intronic defect in a heterozygous state (rs17028, c.1066-25G>A), for which there are no data in ClinVar (https://www.ncbi.nlm.nih.gov/snp/rs372571769#clinical_significance accessed on 21 March 2024). Both sisters displayed chronic respiratory disease, and the older sister also exhibited granulomatous disease. Considering that both defects are located in intron 4, far away from the exon–intron boundary of the 5th exon, we classified them as possible non-pathogenic defects in Table 2.

### 2.3. Association of SERPINA1 Defects with the Clinical Manifestations of PADs

As presented in Table 3, patients that carried heterozygous pathogenic defects of the *SERPINA1* gene displayed clinical manifestations related to AATD. In particular, the presence of the Z allele was significantly associated with the development of chronic liver disease (odds ratio: 29.57, *p* = 0.006). Thus, among the three heterozygous patients for the Z allele, two developed chronic liver disease; the first patient displayed cirrhosis after HBV infection, and the other developed cirrhosis and hypersplenism possibly due to granulomatous disease (established by lymph node biopsy since a liver biopsy was not feasible due to severe thrombocytopenia and coagulopathy). The third carrier of the Z allele was a newly diagnosed 15-year-old patient, without a confirmed liver disease. Two additional patients with pathogenic defects (Table 3) were mentioned with liver disease; the first patient carried a TTC codon (amino acid 76) deletion, developing cirrhosis of unknown origin (since a liver biopsy was not diagnostic) and hypersplenism due to portal hypertension. The second patient carried the p.Leu23Gln missense mutation (rs1379209512, Table 3) with elevated levels of transaminases of unknown etiology.

Regarding respiratory complications, we observed that three of ten patients with pathogenic *SERPINA1* defects developed chronic pulmonary complications; two patients developed bronchiectasis and one developed CRPD (Table 3). However, the presence of pathogenic *SERPINA1* defects was not significantly associated with chronic pulmonary disease.

Finally, patients with pathogenic *SERPINA1* defects exhibited a reduced probability of developing autoimmune disease (odds ratio: 0.17, *p*-value = 0.03) compared to those without pathogenic defects.

## 3. Discussion

Our study suggests that *SERPINA1* defects may affect CVID clinical phenotype, since several genetic defects, including the common Z allele, were associated with liver disease. Although the number of patients analyzed and the number of carriers of *SERPINA1* defects were low, our results represent an initial step for further studies, so that the possible contribution of *SERPINA1* defects as predictors of CVID phenotype and severity may be confirmed.

In 1963, Laurell and Eriksson discovered that serum protein electrophoreses of several individuals with severe COPD of early onset lacked a band for alpha-1 globulin [23], later known as AAT. Six years later, Sharp et al. described an association between AATD and cirrhotic liver disease [24]. Thereafter, numerous AAT glycoforms have been documented by the relative speed of protein migration on gel electrophoresis using isoelectric focusing (IEF), with letters assigned to each variant in alphabetical order [25]. The most common non-pathogenic AAT variant migrates a moderate distance and is designated as the M allele, including nine different glycoforms of the M-AAT protein (subtypes M0–M8) [26]. Conversely, the most prevalent deficiency alleles are designated as S (c.863A>T, p.Glu288Val, rs17580) and Z (c.1096G>A, p.Glu366Lys, rs28929474), and their prevalence in Caucasian populations ranges from 1% to 5%; while some individuals inherit rare pathogenic alleles resulting in either the absence of circulating AAT (null alleles) or poor AAT secretion from hepatocytes (deficiency alleles) or even from a modified inhibitory activity (dysfunctional alleles) [27,28]. The presence of null alleles (denoted as Q0) had no liver inclusions produced as a rule, and no liver disease developed in homozygotes [14,28].

Interestingly, the Z allele is the most common pathogenic allele, resulting in the accumulation of abnormal AAT as inclusions in the rough endoplasmic reticulum of the liver [29]. Homozygotes for the Z allele (Pi*ZZ) display very low levels of circulating AAT leading to early-onset COPD, while liver AAT inclusions predispose to juvenile hepatitis, cirrhosis and hepatocellular carcinoma [29]. Conversely, while the S allele results in lower AAT levels it is not associated with any pulmonary sequelae and liver inclusions but in combination with the Z or other pathogenic (deficiency, dysfunctional or null) alleles leads to AATD [14,29].

In our study, we observed that CVID patients carrying the Z allele in a heterozygous state, along with patients with other pathogenic variants (p.Leu23Gln and p.Phe76del), displayed chronic liver disease, as mentioned above and presented in detail in Table 3. Our findings were in accordance with other studies in the literature, indicating that the Pi*Z allele could be considered a disease modifier for liver disease and stiffness among individuals with obesity and diabetes mellitus [30,31], as well as a risk factor for cirrhosis development in patients with non-alcoholic fatty liver disease (NAFLD) and alcohol misuse [32].

Our findings were in contrast to those from Fazlollahi et al., where no association between the presence of the Z allele and liver disease was identified in Iranian patients with PAD [18]. However, Fazlollahi et al. enrolled only 40 PAD patients including 24 with CVID, and only 2 patients carried the Z allele with no liver disease [18]. Moreover, we did not confirm the findings of the study of Sansom et al., where a higher prevalence of the Z allele was found in 70 CVID patients with bronchiectasis; however, the authors did not provide data for liver disease, while they also considered their findings as inconclusive due to the low number of patients analyzed [16].

The most common *SERPINA1* defect identified in our cohort was rs61761869 (c.1177C>T, p.Pro393Ser). This variant is derived from the M1A allele and results in intracellular proteolysis of AAT, as indicated by the study of Hofker et al. [33]. On the other hand, the p.Phe76del (rs775982338) defect is derived from the M2 allele and results in AAT polymerization, similar to the effect of the Z allele [34]. The effect of the aforementioned *SERPINA1* defects in AAT protein may explain the different clinical phenotype of CVID patients who carry them, with patients carrying the Z or p.Phe76del alleles displaying liver disease, while carriers of the p.Pro393Ser allele do not. Moreover, we found a patient who carried the p.Leu23Gln allele and suffered from bronchiectasis and elevated liver enzymes (Table 3); however, no data for this variant are available from the literature and ClinVar (https://www.ncbi.nlm.nih.gov/snp/rs1379209512#clinical_significance accessed on 21 March 2024).

Interestingly, in our study we observed that PAD patients with pathogenic *SERPINA1* defects exhibit a significantly reduced probability of developing autoimmune disease compared to those without pathogenic defects. As mentioned above, recent studies suggest that AAT has immunomodulatory effects [15], however, the contribution of *SERPINA1* defective variants in autoimmunity is thoroughly obscure. Clearly, further studies should clarify our preliminary findings.

## 4. Materials and Methods

### 4.1. Ethical Statement

Written informed consent was obtained from all participants or an accompanying relative, for a few patients whose consent was not legally applicable (e.g., children). The study was designed according to Helsinki II Declaration ethics and approved by the Ethical Committee of the Faculty of Medicine, University of Thessaly, Greece under the Graduate Study Program “Clinical Applications of Molecular Medicine” (approval code: 1385/5.10.18 and approval date: 18 October 2018).

### 4.2. Patient Characteristics

A total of 80 patients (male/female: 36/44, median age at analysis: 45.0 years, range: 14–71) derived from outpatient clinics of referral centers of primary immunodeficiencies in Greece (University Hospital of Larissa and Evaggelismos General Hospital of Athens) were retrospectively enrolled in the study. Among them, 70 patients (male/female: 31/39, median age at diagnosis: 37.5 years, range: 4–60; median age at analysis: 44.5 years, range: 14–70) fulfilled the classical diagnostic criteria of CVID: (a) low serum levels of IgG, IgA and/or IgM, greater than two standard deviations below the normal mean for their age; (b) poor responses to vaccines, especially polysaccharide ones; (c) exclusion of other defined causes of hypogammaglobulinemia and/or other types of IEI [4,35]. A 44-year-old male patient (age at diagnosis: 12 years) displayed combined IgA and IgG subclass deficiencies with a CVID-like clinical phenotype; two patients (male/female: 1/1, median age at diagnosis: 18.5 years, median age at analysis: 26.0 years, range: 24–28) had an initial diagnosis of CVID, but genetic analysis revealed the presence of pathogenic *CTLA4* mutations [36]. Finally, seven patients displayed mild to moderate hypogammaglobulinemia with recurrent infections and a negative work-up for secondary immunodeficiencies, but did not fulfill the CVID diagnostic criteria, displaying, for example, appropriate immune responses after vaccination (male/female: 3/4, median age at diagnosis: 51.0 years, range: 27–70; median age at analysis: 45.0 years, range: 28–71).

Recorded parameters included demographics, disease symptoms and clinical manifestations (including infections, autoimmunity, lymphoproliferation, granulomatous disease, etc.); specific attention was given to recorded complications due to infections (bronchiectasis, chronic obstructive and/or restrictive respiratory disease) and the presence or absence of hepatic disease, considering the aforementioned principal manifestations due to AATD.

### 4.3. Molecular Analysis

Genomic deoxyribonucleic acid (DNA) was extracted from peripheral blood using the QIAamp DNA Blood Mini Kit (Qiagen Ltd., Crawley, UK), according to the manufacturer’s instructions. Afterward, a standard polymerase chain reaction (PCR) was performed to amplify all five exons (including exon–intron boundaries) of the *SERPINA1* gene, as detailed in Table 4. Following this, PCR products were purified using a PCR purification kit (Qiagen, Crawley, UK) and subsequently sequenced using an ABI Prism 310 genetic analyzer (Applied Biosystems, Foster City, CA, USA) and a BigDye Terminator DNA sequencing kit (Applied Biosystems, Foster City, CA, USA).

### 4.4. Statistical Analysis

Categorical variables are described with the use of frequency and relative frequency. Categorical data were analyzed using chi-square tests after Yate’s correction or Fisher’s exact test. The analysis of continuous variables was conducted using the Mann−Whitney U test, as the assumption of normal distribution was violated. A 5% significance level was set for all analyses. The analysis was carried out with Statistical Package for the Social Sciences (SPSS) version 29.0 (International Business Machines Corporation (IBM) Corp. Released 2021. IBM SPSS Statistics for Windows, Version 29.0. Armonk, NY, USA: IBM Corp.) and GraphPad Prism Software version 10.1.1 (San Diego, CA, USA).

## 5. Conclusions

Our study demonstrates that CVID patients with defective *SERPINA1* variants may display a higher probability of developing hepatic complications, ranging from elevated liver enzymes to cirrhosis. Consequently, we recommend *SERPINA1* genetic analysis in CVID patients at diagnosis in order to identify those with a higher risk for liver disease. Obviously, due to the small sample size of our study, further studies are necessary in order to confirm our results.

## Figures and Tables

**Table 1:** An overview of clinical manifestations of the patients of the study.

	Total	CVID	CTLA4-Related Immune Dysregulation Syndrome	Combined IgAD and Subclass-IgGD	Hypogammaglobulinemia
No	80	70	2	1	7
Sex (male/female)	36/44	31/39	1/1	1/0	3/4
Age at analysis (median, range)	45.0	44.5	26.0	44.0	45.0
	14–71	14–70	24–28		28–71
***Clinical manifestations***					
Lymphoproliferation * (n, %)	50, 62.5	47, 67.1	1, 50.0	0, 0	2, 28.6
Chronic respiratory disease (n, %)	25, 31.3	22, 31.4	0, 0	1, 100	2, 28.6
CRPD (n, %)	13, 16.3	13, 18.6	0, 0	0, 0	0, 0
COPD (n, %)	7, 8.8	4, 5.7	0, 0	1, 100	2, 28.6
CRPD/COPD (n, %)	5, 6.3	5, 7.1	0, 0	0, 0	0, 0
Bronchiectasis (n, %)	23, 28.8	22, 31.4	0, 0	0, 0	1, 14.3
Liver disease ** (n, %)	10, 12.5	9, 12.9	0, 0	0, 0	1, 14.3
Granulomatous disease (n, %)	10, 12.5	10, 14.3	0, 0	0, 0	0, 0
Autoimmune manifestations ^ (n, %)	44, 55.0	40, 57.1	2, 100	0, 0	2, 28.6
Atopy (n, %)	23, 28.8	21, 30.0	2, 100	0, 0	0, 0
Neoplasia ^^ (n, %)	13, 16/3	9, 12.9	1, 50.0	0, 0	3, 42.8

Abbreviations: CRPD, chronic restrictive pulmonary disease; COPD, chronic obstructive pulmonary disease; CVID, common variable immunodeficiency; IgAD, IgA deficiency; IgGD, IgG deficiency. * Lymphoproliferation includes splenomegaly, lymphadenopathy, gastrointestinal lymph infiltrates; ** liver disease includes nodular regenerative hyperplasia (NRH), granulomatous disease of liver, unexplained elevated transaminase levels and cirrhosis after chronic HBV or HCV infection; ^ autoimmune manifestations include autoimmune hemolytic anemia, autoimmune thrombocytopenic purpura, Evans syndrome, pernicious anemia, thyroid disease, psoriasis, vitiligo, lupus, autoimmune hepatitis, primary biliary cirrhosis, vasculitis, myelitis; ^^ neoplasia includes the development of lymphomas (Hodgkin or/and non-Hodgkin; 5), breast cancer (2), lung cancer (1), uterus cancer (1), stomach cancer (2), colon cancer (1), thyroid cancer (1), acute lymphoblastic leukemia (1).

**Table 2:** *SERPINA1* defects identified in the patients of the study.

No.	Genetic Defect	Heterozygous	Homozygous	Allele Frequency
**Pathogenic defects**				
1	c.1096G>A, p.Glu366Lys, rs28929474 (PI*Z variant)	3	0	1.88%
2	c.863A>T, p.Glu288Val, rs17580 (PI*S variant)	1	0	0.63%
3	c.1177C>T, p.Pro393Thr, rs61761869	4	0	2.50%
4	c.68T>A, p.Leu23Gln, rs1379209512	1	0	0.63%
5	c.226_8delTTC, p.Phe76del, rs77598233	1	0	0.63%
**Non-pathogenic (benign) defects**				
1	c.710T>C, p.Val237Ala, rs6647 (PI*M1A variant)	32	3	23.75%
2	c.374G>A, p.Arg125His, rs709932 (PI*M2/M4 variant)	21	3	16.88%
3	rs1303, c.1200A>C, p.Glu400Asp (PI*M3 variant)	26	4	21.25%
4	c.43C>T, p.Leu15=, rs147283849	4	0	2.50%
5	c.171C>T, p.Phe57=, rs150784949	2	0	1.25%
6	c.424C>T, p.Leu142=, rs20546	3	0	1.88%
7	c.967C>T, p.Leu323=, rs150455534	2	0	1.25%
**Possible non-pathogenic defects**				
1	g.17028G>A, c.1066-25G>A, rs372571769	2	0	1.25%
2	c.1066-87T>C (novel defect)	1	0	0.63%

**Table 3:** Clinical characteristics of CVID patients carrying heterozygous *SERPINA1* defects.

No.	Sex	Age at Diagnosis	Age at Analysis	*SERPINA1* Genetic Defects	Clinical Manifestations/ Complications of the Disease *
1	F	22	50	c.68T>A, p.Leu23Gln, rs1379209512	Bronchiectasis Elevated liver enzymes Hashimoto disease, iridocyclitis (uveitis)
2	F	46	52	c.226_8delTTC, p.Phe76del, rs775982338	Cirrhosis (unknown origin) Uterus cancer Atopy
3	F	30	39	c.863A>T, p.Glu288Val, rs17580, PI*S allele	Atopy
4	M	14	15	c.1096G>A, p.Glu366Lys, rs28929474, PI*Z allele	CRPD Evans syndrome
5	M	4	40	c.1096G>A, p.Glu366Lys, rs28929474, PI*Z allele	Cirrhosis (history of HBV infection)
6	F	29	42	c.1096G>A, p.Glu366Lys, rs28929474, PI*Z allele	Cirrhosis (possibly due to granuloma formation) Granulomatous disease
7	F	32	37	c.1177C>T, p.Pro393Ser, rs61761869	Bronchiectasis
8	F	39	43	c.1177C>T, p.Pro393Ser, rs61761869	No complications (history of recurrent respiratory infections)
9	F	51	63	c.1177C>T, p.Pro393Ser, rs61761869	No complications (history of recurrent gastrointestinal infections)
10	F	58	62	c.1177C>T, p.Pro393Ser, rs61761869	No complications (history of recurrent respiratory infections)

Abbreviations: CRPD, chronic restrictive pulmonary disease; F, female; M, male; HBV, hepatitis B virus. * All patients displayed recurrent infections (mainly respiratory) for several years before diagnosis, while all patients with the exception of patients #3 and #8 also exhibited benign lymphoproliferation (splenomegaly and/or lymphadenopathy and/or intestine lymph infiltrates).

**Table 4:** Primers and PCR conditions used in the study.

Exon	Primers	PCR Conditions	PCR Length
1A	F: 5′-AAGGCTCCTTCCTGTCCAAG-3′ R: 5′-CGCTGCTCTACATCCACTCA-3′	94 °C for 2 min, followed by 32 cycles (94 °C for 30 s, 60 °C for 30 s, 72 °C for 30 s) and a final elongation at 72 °C for 5 min	494 bp
1B	F: 5′-CCATCAAGAGGGTGTTTGTGT-3′ R: 5′-CGGATACCCACTCCACAAC-3′	94 °C for 2 min, followed by 32 cycles (94 °C for 30 s, 60 °C for 30 s, 72 °C for 1 min) and a final elongation at 72 °C for 5 min	676 bp
2	F: 5′-GTACTTGGCACAGGCTGGTT-3′ R: 5′-ATGCATTGCCAAGGAGAGTT-3′	94 °C for 2 min, followed by 32 cycles (94 °C for 30 s, 61 °C for 30 s, 72 °C for 1 min) and a final elongation at 72 °C for 5 min	862 bp
3	F: 5′-GAGGGATGTGTGTCGTCAAG-3′ R: 5′-TAGCAGTGACCCAGGGATGT-3′	94 °C for 2 min, followed by 32 cycles (94 °C for 30 s, 61 °C for 30 s, 72 °C for 30 s) and a final elongation at 72 °C for 5 min	521 bp
4	F: 5′-TAGTGTGGGTGGAGGACACA-3′ R: 5′-CAGCCTGGGTCTTCATTTGT-3′	94 °C for 2 min, followed by 32 cycles (94 °C for 30 s, 60 °C for 30 s, 72 °C for 30 s) and a final elongation at 72 °C for 5 min	397 bp
5	F: 5′-GTGACAGGGAGGGAGAGGAT-3′ R: 5′-CTGTTACCTGGAGCCCACAT-3′	94 °C for 2 min, followed by 32 cycles (94 °C for 30 s, 62 °C for 30 s, 72 °C for 30 s) and a final elongation at 72 °C for 5 min	494 bp

Abbreviations: PCR, polymarase chain reaction; bp, base pair; F, forward; R, reverse.

## Data Availability

The data supporting the findings are available, only for sections non-infringing personal information, from the corresponding author upon reasonable request.
